# Diagnostic Performance of ACR TI-RADS, EU-TI-RADS, and Individual Ultrasound Features for Predicting Thyroid Malignancy: A Histopathology-Validated Analysis of Surgically Excised Nodules

**DOI:** 10.7759/cureus.115751

**Published:** 2026-09-03

**Authors:** Abdelwahed S Abougazia, Amal Mahran, Ban Abdullah, Ahmed Abdelmoneim, Maryam M Alemadi, Soliena Alnakawa, Fisal Haritani, Rana Bakkar, Hussameddin Alali, Abdulla Alnaama, Maysaa Ashkanani, Ahmed Sameer Alnuaimi, Tamer Ali

**Affiliations:** 1 Radiology, Primary Health Care Corporation (PHCC), Doha, QAT; 2 Clinical Imaging, Hamad Medical Corporation, Doha, QAT; 3 Family Medicine, Primary Health Care Corporation (PHCC), Doha, QAT; 4 Clinical Research, Primary Health Care Corporation (PHCC), Doha, QAT

**Keywords:** acr ti-rads, diagnostic accuracy, diagnostic performance, eu-ti-rads, follicular thyroid carcinoma, histopathology, thyroid malignancy, thyroid nodules, thyroid ultrasonography, ultrasound

## Abstract

Objective: The primary objective was to compare the histopathology-validated diagnostic performance of the American College of Radiology Thyroid Imaging Reporting and Data System (ACR TI-RADS) and the European Thyroid Imaging Reporting and Data System (EU-TI-RADS) for predicting malignancy in surgically excised thyroid nodules. Secondary objectives were to evaluate the discriminatory contribution of individual ultrasound features, characterize threshold-specific classification errors, examine performance by malignant histological subtype, and assess the potential influence of within-patient clustering.

Methods: In this retrospective secondary analysis of an institutional review board-approved cohort, 284 surgically excised thyroid nodules from 233 patients (170 female, 73.0%; mean age: 43.5 ± 10.9 years) were analysed using postoperative histopathology as the reference standard. Nodules were categorized using ACR TI-RADS and EU-TI-RADS. Diagnostic performance was assessed using receiver operating characteristic (ROC) analysis and threshold-based 2×2 contingency tables. Sensitivity, specificity, accuracy, positive predictive value (PPV), and negative predictive value (NPV) were reported as n/N (%), with 95% confidence intervals. Confusion matrix analysis was performed to evaluate true-positive, false-positive, true-negative, and false-negative classifications. Because some patients contributed more than one nodule, the potential influence of within-patient clustering was evaluated using cluster-size characterization and a single-index-nodule sensitivity analysis. Multivariable regression modelling and formal interobserver agreement analysis were not performed and are addressed in the Limitations section.

Results: Histopathology identified 150/284 malignant nodules (52.8%) and 134/284 benign nodules (47.2%). ACR TI-RADS showed the highest discriminatory performance (AUC of 0.950 for point score and 0.948 for categories), followed by EU-TI-RADS (AUC of 0.933). At clinically relevant thresholds, ACR TI-RADS TR4 or higher identified 131/150 malignant nodules and correctly classified 127/134 benign nodules, yielding sensitivity 87.3%, specificity 94.8%, and accuracy 90.8%. EU-TI-RADS category 4 or higher identified 130/150 malignant nodules and correctly classified 130/134 benign nodules, yielding a sensitivity of 86.7%, a specificity of 97.0%, and an accuracy of 91.5%. ACR TI-RADS produced slightly fewer false-negative classifications, whereas EU-TI-RADS produced fewer false-positive classifications.

Conclusions: ACR TI-RADS and EU-TI-RADS both demonstrated excellent histopathology-validated diagnostic performance in this surgically enriched cohort. Differences between the systems mainly reflected distinct classification error profiles rather than clear superiority of one system. Feature-level and confusion matrix analyses provided additional clinically relevant information, but the findings should be interpreted in the context of surgical-selection bias and may not be directly generalizable to unselected thyroid nodule populations. Because this was a surgically enriched cohort with a malignancy prevalence of 52.8%, predictive values should not be extrapolated to unselected screening or surveillance populations. Sensitivity, specificity, and AUC may also differ in unselected populations because of spectrum and verification bias. Prospective multicenter validation is required.

## Introduction

Thyroid nodules are common in clinical practice and require structured evaluation because a subset is malignant and necessitates further investigation and management [1-3]. Ultrasound is central to thyroid nodule assessment as it provides non-invasive characterization of composition, echogenicity, margins, shape, orientation, and echogenic foci, all of which are essential for malignancy risk stratification [2,3]. Individual suspicious sonographic features, including marked hypoechogenicity, punctate echogenic foci, irregular margins, and taller-than-wide orientation, have long been associated with malignancy; however, their diagnostic value varies when assessed in isolation, and no single feature is sufficiently reliable alone [2-4].

To improve diagnostic consistency and reduce unnecessary biopsy, several structured ultrasound-based risk stratification systems have been developed, most notably the American College of Radiology Thyroid Imaging Reporting and Data System (ACR TI-RADS) and the European Thyroid Association EU-TI-RADS [5,6]. These systems integrate multiple sonographic characteristics into standardized categories to estimate malignancy risk and guide fine-needle aspiration decisions [5,6]. Comparative studies and recent meta-analyses have consistently demonstrated that both ACR TI-RADS and EU-TI-RADS provide clinically useful risk stratification, although differences exist in sensitivity, specificity, category distribution, and biopsy-reduction performance across populations and study designs [7-12].

Despite the widespread adoption of TI-RADS frameworks, relatively less emphasis has been placed on the individual contribution of specific ultrasound features when histopathology is used as the reference standard [4,7]. This issue is clinically relevant because some sonographic features are diagnostically informative but show interobserver variability in routine practice, particularly in the assessment of nodule margins [13]. In addition, histology-correlated evidence has shown that a subset of benign nodules may exhibit suspicious ultrasound patterns and be categorized as high-risk TI-RADS lesions, thereby reducing specificity when ultrasound findings are interpreted without integrated clinical and pathological correlation [14].

Although several studies have compared ACR TI-RADS and EU-TI-RADS, including analyses based on histopathological reference standards, differences in reported diagnostic performance remain influenced by study design, population characteristics, and risk-category distribution. Relatively fewer studies have incorporated feature-level discrimination analyses, explicit evaluation of classification error profiles, and methodological approaches that account for within-patient clustering in surgically treated cohorts. This work complements our previously published PHCC cohort study of 809 biopsied nodules, which evaluated guideline-based fine-needle aspiration recommendations using a composite reference standard [15]. The present analysis is a distinct secondary study restricted to the 284 surgically excised nodules within that programme, uses postoperative histopathology exclusively as the reference standard, and addresses different research questions: intrinsic feature-level discrimination, confusion-matrix error profiling, assessment of the potential influence of within-patient clustering, and histological subtype-specific performance. The present study includes a surgically excised subset of participants from the previously published cohort; therefore, the underlying source population overlaps. However, the present analysis uses a different analytic cohort, an exclusively postoperative histopathological reference standard, distinct research questions, and newly generated analyses, tables, and figures. The relationship between the two studies is disclosed transparently, and the previous publication is cited.

The primary objective was to compare the histopathology-validated diagnostic performance of ACR TI-RADS and EU-TI-RADS for predicting malignant versus benign postoperative histopathology in surgically excised thyroid nodules. Secondary objectives were to evaluate the discriminatory contribution of individual ultrasound features, characterize threshold-specific false-positive and false-negative classifications, examine diagnostic performance by malignant histological subtype, and assess the potential influence of within-patient clustering.

## Materials and methods

Study design and setting

This retrospective diagnostic accuracy study used data from thyroid nodules referred from primary care clinics and tertiary referral hospitals within a national healthcare system and restricted the analysis to surgically treated nodules with definitive postoperative histopathology. This study represents a secondary analysis of a previously approved retrospective cohort of patients with thyroid nodules evaluated at Primary Health Care Corporation (PHCC), Qatar. The original dataset included patients who underwent thyroid ultrasound and fine-needle aspiration cytology between November 2023 and November 2024. The present analysis was limited to surgically excised nodules with available histopathological confirmation, which served as the reference standard for diagnostic performance evaluation. The decision to proceed to surgery was made independently of this study according to routine clinical pathways. Surgical indications included suspicious or indeterminate cytology, sonographic suspicion, compressive or cosmetic symptoms, multinodular disease, patient preference, and multidisciplinary clinical recommendations. For nodules ultimately shown to be benign on postoperative histopathology, surgery may have been undertaken because of indeterminate or discordant preoperative findings, compressive or cosmetic symptoms, multinodular disease, patient preference, or a multidisciplinary recommendation. Individual surgical indications were not systematically coded in the analytic dataset; therefore, their relative frequencies could not be quantified. Nodules were included consecutively within the study window. There were no missing key ultrasound descriptors in the final analytic dataset. Sample-size calculation was not performed because this was a secondary analysis of an existing cohort.

Relationship to prior publication

This study represents a secondary analysis of the same PHCC thyroid-nodule programme previously reported by Abougazia et al. [15]. The previous study evaluated 809 nodules with definitive outcomes and focused on the performance of guideline-based fine-needle aspiration recommendations using a composite reference standard. The current study includes the surgically excised subset of that source cohort and therefore has participant-level and source-data overlap. However, it is restricted to 284 nodules with matched postoperative histopathology and addresses distinct research questions, including histopathology-validated discrimination, feature-level performance, classification-error profiles, histological subtype-specific performance, and within-patient clustering. No tables, figures, receiver operating characteristic (ROC) curves, or diagnostic performance estimates from the previous publication are reproduced in the present manuscript.

Ethical approval was obtained from the institutional review board, with waiver of informed consent because anonymized retrospective clinical data were used. The study was conducted under Institutional Review Board approval (BUHOOTH-D-24-00087). The current investigation represents a secondary analysis of data collected under the approved protocol.

Patients and eligibility criteria

Inclusion criteria: The study included 284 surgically excised thyroid nodules from 233 patients. Only nodules with both complete preoperative ultrasound variables required for TI-RADS classification and matched postoperative histopathology were included. The nodule was used as the primary unit of analysis because TI-RADS classification, ultrasound feature assessment, and histopathologic verification were assigned at the nodule level. In patients with multiple surgically excised nodules, a maximum of two nodules per patient was included.

Exclusion criteria: Nodules were excluded from the histopathology-validated analysis when any of the following applied: (i) prior thyroid surgery, radiofrequency ablation, or ethanol sclerotherapy affecting the same nodule; (ii) recurrent or metastatic thyroid carcinoma involving the same nodule bed; (iii) technically inadequate ultrasound images precluding TI-RADS classification; (iv) missing key ultrasound descriptors required for ACR TI-RADS point calculation; and (v) unmatched or ambiguous surgical-pathology correlation. The final analytic dataset comprised 284 nodules from 233 patients.

Ultrasound acquisition and interpretation: Ultrasound examinations were performed by board-certified radiologists and experienced sonographers (median clinical experience ≥ 5 years) across primary care centres and tertiary referral hospitals within the national healthcare system. Studies were acquired on high-resolution linear-array transducers operating at 5-15 MHz according to standard institutional thyroid ultrasound protocols, with grayscale, colour Doppler, and power Doppler cine loops archived to the institutional Picture Archiving and Communication System (PACS).

For the study analysis, all archived images were independently reassessed by three consultant radiologists, each with at least 10 years of dedicated thyroid-imaging experience. Reviewers were blinded to the original clinical report, cytology, and postoperative histopathology at the time of image reassessment. Each reader initially assigned ultrasound descriptors and TI-RADS classifications independently. Disagreements were subsequently resolved by joint consensus review. Formal interobserver agreement statistics were not calculated because individual reader-level classifications were not retained in the final analytic dataset.

Index tests

Each thyroid nodule was classified according to the ACR TI-RADS using the total point score and corresponding TR category, as defined in the original ACR TI-RADS white paper [5]. Each nodule was also classified according to the EU-TI-RADS, using the pattern-based definitions and categorization rules specified in the original EU-TI-RADS guideline [6]. ACR TI-RADS point scores were calculated by summing the points assigned for composition, echogenicity, shape or orientation, margins, and echogenic foci. The total score was subsequently converted into the corresponding ACR TI-RADS category from TR1 to TR5. EU-TI-RADS categories were assigned from category 2 to category 5 based on the sonographic pattern and the presence of high-suspicion ultrasound features.

The index-test classifications used in the statistical analyses were based on the consensus assessments obtained after independent blinded image reassessment by the three consultant radiologists and resolution of disagreements through joint review. The reviewers were blinded to cytological and postoperative histopathological outcomes when assigning the ultrasound descriptors and TI-RADS classifications.

Diagnostic discrimination was evaluated for the continuous ACR TI-RADS point score, the ordinal ACR TI-RADS categories, and the ordinal EU-TI-RADS categories. Threshold-based diagnostic performance was evaluated using prespecified, clinically relevant category thresholds: ACR TI-RADS TR4 or higher and EU-TI-RADS category 4 or higher were classified as test-positive, while categories below these thresholds were classified as test-negative. These thresholds were used for binary malignancy prediction and classification-error analysis and should not be interpreted as size-independent fine-needle aspiration thresholds.

The diagnostic contribution of individual ACR TI-RADS component criteria and selected additional grayscale and Doppler ultrasound features was also evaluated. Feature-level ROC analyses were conducted using the original categorical or ordinal coding of each ultrasound variable. The category-level cross-tabulations (Appendix, Supplementary Tables S2 and S3) permit independent reconstruction of the principal category-based ROC and threshold-specific diagnostic performance analyses.

Reference standard

Surgical histopathology served as the reference standard and classified nodules as benign or malignant. Histopathology reports were matched to the corresponding preoperative ultrasound nodule using anatomic location, laterality, nodule size, operative documentation, and pathology description when multiple nodules were present. Malignant nodules were further stratified into papillary thyroid carcinoma and other malignancies for subgroup analyses.

Statistical analysis

The study included consecutive eligible surgically excised nodules identified during the study period. No imputation procedures were performed because all nodules included in the final analytic dataset had complete ultrasound variables required for TI-RADS classification and matched postoperative histopathology. Diagnostic discrimination was assessed using ROC analysis, with calculation of the area under the curve (AUC) and corresponding 95% confidence intervals (CI) for ACR TI-RADS point score, ACR TI-RADS categories, EU-TI-RADS categories, and selected individual ultrasound features. ROC analysis of individual ultrasound features (Appendix, Supplementary Table S7) was performed using the original ordinal categorical coding of each variable. Threshold-based diagnostic performance was calculated from observed 2×2 contingency tables and reported as n/N (%) with 95% CI, including sensitivity, specificity, positive predictive value (PPV), negative predictive value (NPV), and overall accuracy. Confidence intervals for proportions were calculated using the Wilson score method. Positive and negative likelihood ratios, diagnostic odds ratios, and the Youden index were also reported. Confusion matrix analysis was performed to display true-positive, false-positive, true-negative, and false-negative classifications for clinically relevant cutoffs (ACR TI-RADS TR4 or higher and EU-TI-RADS ≥ 4). Because some patients contributed more than one thyroid nodule, potential within-patient clustering was evaluated using two complementary approaches. First, the cluster-size distribution was characterized descriptively; the maximum cluster size was two nodules per patient (Appendix, Supplementary Table S8). Second, a single-index-nodule sensitivity analysis was performed by restricting the dataset to one nodule per patient. For patients with multiple nodules, the index nodule was selected according to the highest TI-RADS category, and, if categories were tied, the largest maximum diameter (Appendix, Supplementary Table S9). Because the single-index-nodule sensitivity analysis yielded diagnostic performance estimates within approximately 1.2 percentage points of the primary nodule-level estimates, unclustered Wilson score confidence intervals were retained for the primary analyses. A paired ROC comparison between ACR TI-RADS and EU-TI-RADS was performed using bootstrap resampling with 2,000 replicates to estimate the difference between AUCs (ΔAUC) and its 95% CI. Statistical significance was defined as a two-sided P value < 0.05. Multivariable logistic regression was not performed. Cross-tabulation demonstrated that microcalcifications, taller-than-wide orientation, very hypoechoic echotexture, and irregular or lobulated margins were absent in all 134 benign nodules but present in subsets of malignant nodules, producing zero benign cells and quasi-complete separation (Appendix, Supplementary Table S11). Under these conditions, conventional multivariable logistic-regression modelling would yield unstable or non-finite coefficient estimates and adjusted odds ratios. Feature-level discrimination was therefore evaluated using univariate ROC analysis, while integrated risk stratification was assessed using the composite ACR TI-RADS and EU-TI-RADS classifications.

Formal interobserver agreement statistics, including kappa coefficients or intraclass correlation coefficients, were not calculated because individual reader-level classifications were not retained in the final analytic dataset. The analysis therefore used the consensus classifications obtained after independent blinded reassessment and resolution of disagreements by joint review; this is addressed as a limitation. Descriptive and diagnostic performance analyses were performed using Statistical Product and Service Solutions (SPSS, version 29.0; IBM SPSS Statistics for Windows, Armonk, NY). Additional subgroup and feature-level analyses are provided in the Appendix.

Reporting standards

The Standards for Reporting Diagnostic Accuracy Studies (STARD) framework was used to guide reporting of participant selection, index test interpretation, reference standard definition, flow of included and excluded nodules, and diagnostic accuracy estimates [16], with the study flow illustrated in Figure 1.

**Figure 1:**
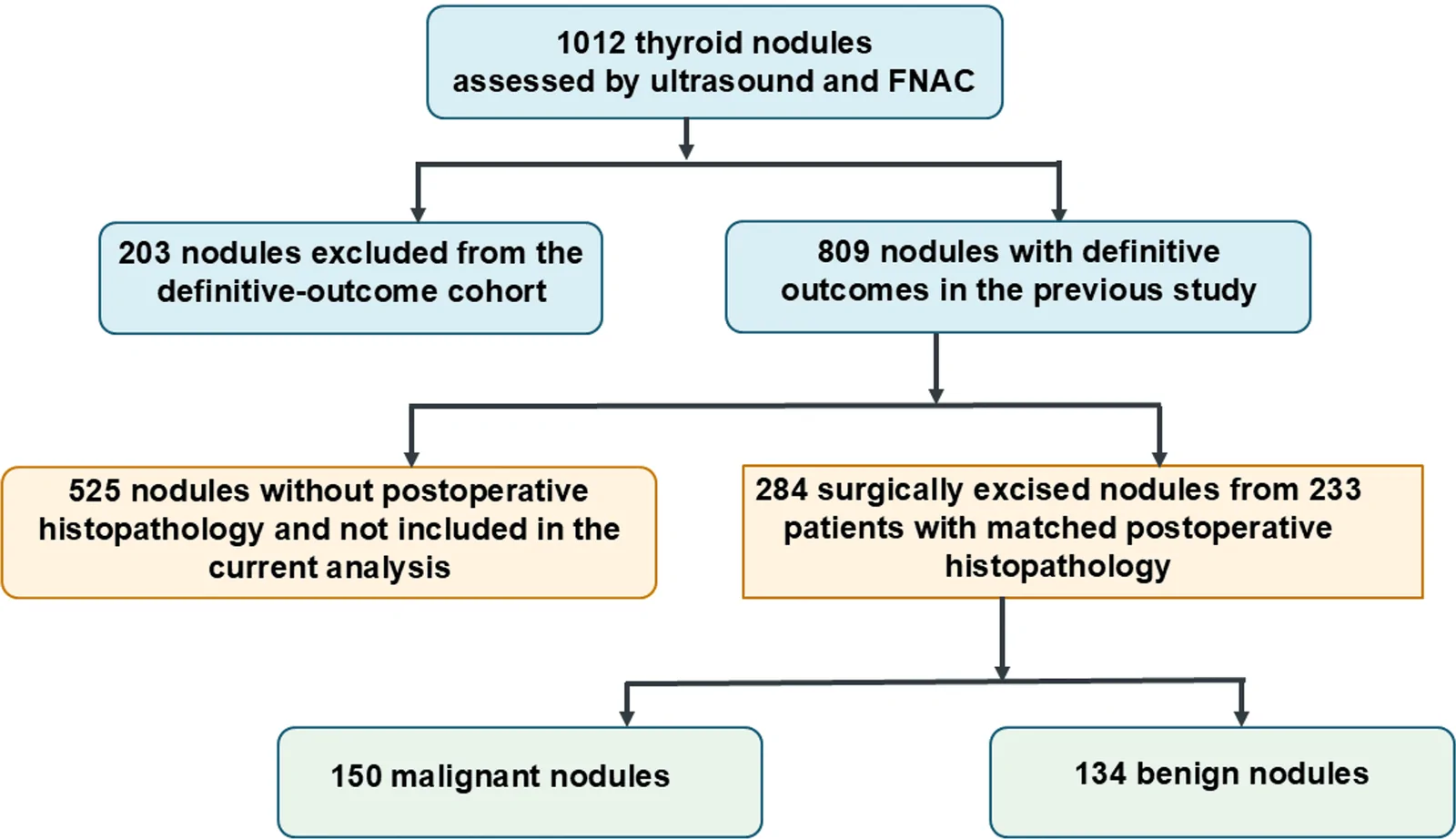
Study flow diagram illustrating the selection of the histopathology-validated surgical cohort A total of 1,012 thyroid nodules underwent ultrasound assessment and fine-needle aspiration cytology. Of these, 809 met the definitive-outcome criteria of the previously published cohort, while 203 were excluded. The present secondary analysis was restricted to 284 surgically excised nodules from 233 patients with matched postoperative histopathology, comprising 150 malignant and 134 benign nodules. The remaining 525 nodules were not included in the current surgical-histopathology analysis. FNAC = fine-needle aspiration cytology

## Results

Study population and baseline characteristics

A total of 284 surgically excised thyroid nodules from 233 patients were included in the analysis. The included patients had a mean age of 43.5 ± 10.9 years (median: 43, interquartile range: 36-51, range: 19-80), and 170 of 233 (73.0%) were female. One hundred and eighty-two patients (78.1%) contributed one operated nodule, and 51 patients (21.9%) contributed two operated nodules, yielding the final analytic set of 284 nodules. Histopathology classified 150 nodules (52.8%) as malignant and 134 (47.2%) as benign, with papillary thyroid carcinoma accounting for most malignant cases (121/150). Baseline demographic and ultrasound characteristics are summarized in Table 1, while detailed ultrasound feature distributions and TI-RADS category cross-tabulations are provided in the Appendix (Supplementary Tables S1-S4).

**Table 1:** Baseline demographic and ultrasound characteristics Patient-level characteristics were calculated on 233 unique patients identified by the case-sensitive HC number field. Fifty-one patients contributed two surgically excised nodules each; the first occurrence of each patient in the operated-cohort dataset was used to derive age and sex. Nodule-level characteristics were calculated on all 284 surgically excised nodules. Percentages for nodule-level rows are calculated using n = 284; percentages for patient-level rows are calculated using n = 233. Patient-level characteristics (age, sex) are reported for the 233 included patients; nodule-level ultrasound characteristics are reported for the 284 surgically excised nodules. Values are presented as n (%) unless otherwise indicated. Age is reported as mean ± standard deviation, with median and interquartile range (IQR) in parentheses.

Characteristic	Value
Patient-level characteristics (n = 233)	
Age (years), mean ± SD	43.5 ± 10.9
Age (years), median (IQR) (range)	43 (36-51) (19-80)
Female	170 (73.0%)
Male	63 (27.0%)
Patients contributing 1 nodule	182 (78.1%)
Patients contributing 2 nodules	51 (21.9%)
Nodule-level characteristics (n = 284)	
Nodule site	
Right lobe	130 (45.8%)
Left lobe	136 (47.9%)
Isthmus	18 (6.3%)
Maximum size category	
0.5-<1.0 cm	14 (4.9%)
1.0-<1.5 cm	47 (16.5%)
1.5-2.0 cm	28 (9.9%)
>2.0-<2.5 cm	20 (7.0%)
≥2.5 cm	175 (61.6%)
Composition	
Solid	120 (42.3%)
Mixed cystic - solid	101 (35.6%)
Almost entirely solid	56 (19.7%)
Almost entirely cystic	5 (1.8%)
Cystic	2 (0.7%)
Echogenicity	
Isoechoic	161 (56.7%)
Hypoechoic	101 (35.6%)
Very (markedly) hypoechoic	12 (4.2%)
Anechoic	7 (2.5%)
Hyperechoic	3 (1.1%)
Shape	
Oval	268 (94.4%)
Irregular	11 (3.9%)
Round	5 (1.8%)
Orientation	
Wider-than-tall	268 (94.4%)
Taller-than-wide	16 (5.6%)
Margins	
Smooth	150 (52.8%)
Ill-defined	76 (26.8%)
Lobulated	30 (10.6%)
Irregular	28 (9.9%)
Echogenic foci/calcifications	
Microcalcifications present	95 (33.5%)
Macrocalcifications present	42 (14.8%)
Rim calcifications present	7 (2.5%)
Extrathyroidal extension	
Absent	280 (98.6%)
Capsular bulge	4 (1.4%)
Overt extension	0 (0.0%)
Doppler vascularity	
Type II (peri- and/or slight intranodular flow)	174 (61.3%)
Type III (marked intranodular flow)	66 (23.2%)
Type I (no peri-/intranodular flow)	24 (8.5%)
Not assessed / NA	20 (7.0%)
Halo sign	
Absent	267 (94.0%)
Present	17 (6.0%)
Comet-tail artifact	
Absent	253 (89.1%)
Present	31 (10.9%)

ROC analysis (overall diagnostic performance)

ACR TI-RADS demonstrated a numerically higher AUC than EU-TI-RADS (0.948 versus 0.933), corresponding to an absolute difference (ΔAUC) of approximately 0.015. Paired bootstrap resampling (2,000 replicates) yielded a mean ΔAUC of approximately 0.015, with a 95% confidence interval of approximately −0.002 to 0.033. Because the confidence interval crossed zero, no statistically significant difference in overall discrimination was demonstrated between the two systems. ROC curves comparing the performance of both systems are presented in Figure 2 (Table 2).

**Figure 2:**
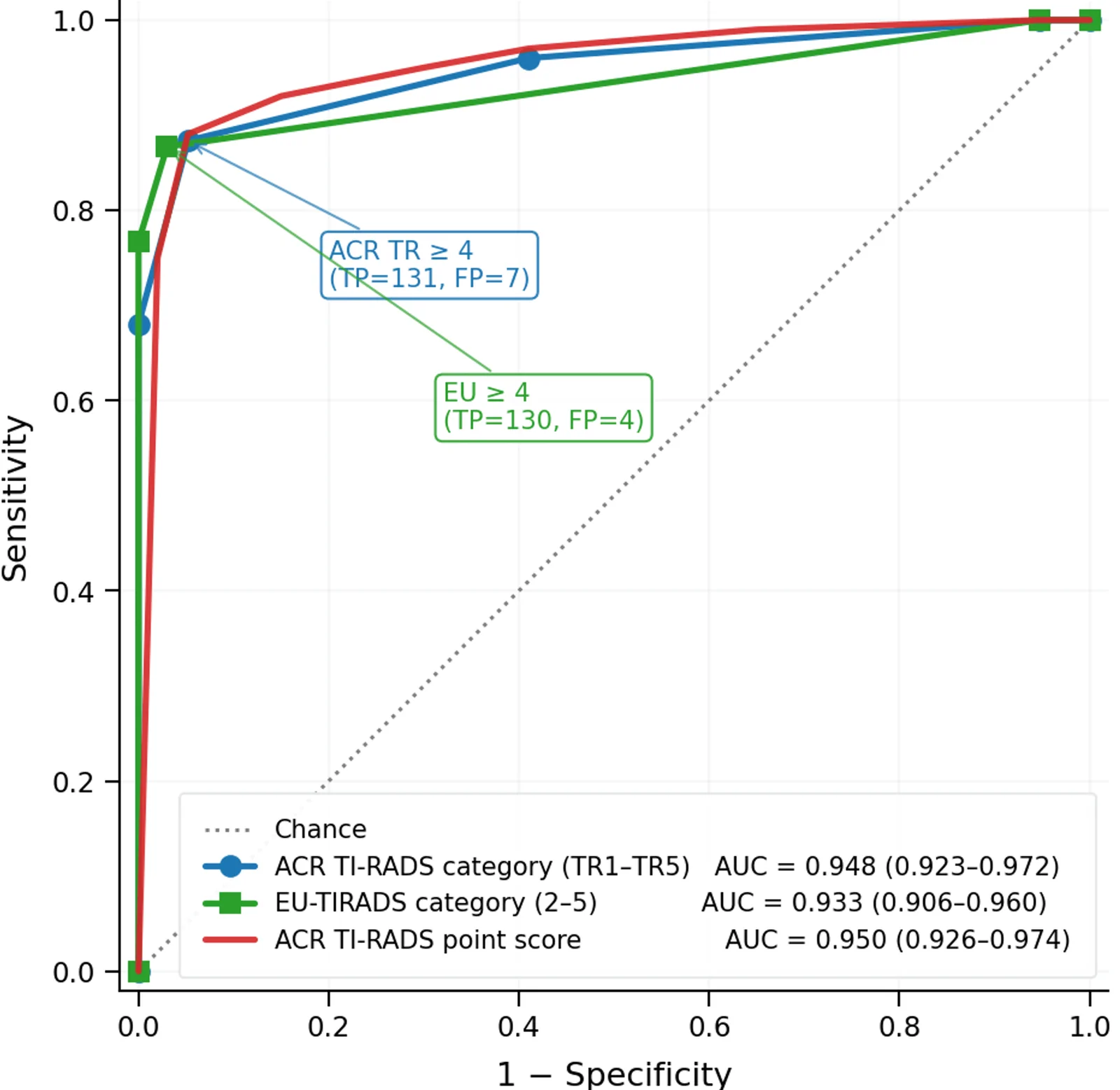
Receiver operating characteristic curves comparing ACR TI-RADS point score, ACR TI-RADS category (TR1-TR5), and EU-TI-RADS category (2-5) for predicting malignant versus benign thyroid nodules in a surgically excised cohort (n = 284; 150 malignant and 134 benign), using histopathology as the reference standard Areas under the curve with 95% confidence intervals were 0.950 (0.926-0.974) for the ACR TI-RADS point score, 0.948 (0.923-0.972) for ACR TI-RADS categories, and 0.933 (0.906-0.960) for EU-TI-RADS categories. Category-level curves were computed empirically from the cross-tabulations in the Appendix (Supplementary Tables S2 and S3). The clinical thresholds ACR TR4 or higher and EU-TI-RADS category 4 or higher are annotated on the corresponding curves. The dashed diagonal line represents chance performance. ACR TI-RADS = American College of Radiology Thyroid Imaging Reporting and Data System; EU-TI-RADS = European Thyroid Imaging Reporting and Data System

**Table 2:** Diagnostic performance (ROC analysis) of ultrasound risk stratification systems for predicting malignant versus benign histopathology in surgically excised thyroid nodules (n = 284; 150 malignant, 134 benign) AUC values with 95% confidence intervals (CI) and P values are reported. ACR TI-RADS = American College of Radiology Thyroid Imaging Reporting and Data System; EU-TI-RADS = European Thyroid Imaging Reporting and Data System

Measure	AUC	95% CI	P value
ACR TI-RADS point score	0.95	0.926-0.974	<0.001
ACR TI-RADS categories	0.948	0.923-0.972	<0.001
EU-TI-RADS categories	0.933	0.906-0.960	<0.001

Patient-level sensitivity analysis yielded diagnostic performance estimates that were nearly identical to those obtained from the primary nodule-level analysis. No clinically meaningful differences were observed in sensitivity, specificity, predictive values, or overall accuracy, indicating that inclusion of multiple nodules from the same patient did not materially influence the threshold-based findings. Although the ACR TI-RADS point score yielded the numerically highest AUC, the absolute differences between the three approaches were small. The ROC curves showed substantial overlap, indicating broadly similar discriminatory performance across the systems. These findings suggest that the observed differences in AUC should be interpreted as modest numerical variations rather than evidence of a clinically meaningful difference in overall diagnostic discrimination. The slightly higher AUC observed for the ACR TI-RADS point score likely reflects the greater granularity of the continuous scoring system compared with category-based classification. Nevertheless, both ACR TI-RADS and EU-TI-RADS demonstrated excellent ability to distinguish malignant from benign nodules in this histopathology-validated surgical cohort.

Threshold-based diagnostic performance

At clinically relevant thresholds, both systems showed high and balanced diagnostic performance (Table 3). ACR TI-RADS TR4 or higher identified 131/150 malignant nodules and correctly classified 127/134 benign nodules, yielding a sensitivity of 87.3% (95% CI: 81.1%-91.7%), a specificity of 94.8% (95% CI: 89.6%-97.4%), PPV of 94.9% (95% CI: 89.9%-97.5%), NPV of 87.0% (95% CI: 80.6%-91.5%), and an accuracy of 90.8% (95% CI: 86.9%-93.7%). EU-TI-RADS category 4 or higher identified 130/150 malignant nodules and correctly classified 130/134 benign nodules, yielding a sensitivity of 86.7% (95% CI: 80.3%-91.2%), a specificity of 97.0% (95% CI: 92.6%-98.8%), PPV of 97.0% (95% CI: 92.6%-98.8%), NPV of 86.7% (95% CI: 80.3%-91.2%), and an accuracy of 91.5% (95% CI: 87.7%-94.3%).

**Table 3:** Threshold-based diagnostic performance of ACR TI-RADS and EU-TI-RADS for predicting malignant histopathology in surgically excised thyroid nodules (n = 284) Values are presented as n/N (%), with 95% confidence intervals, unless otherwise indicated. ACR TI-RADS = American College of Radiology Thyroid Imaging Reporting and Data System; EU-TI-RADS = European Thyroid Imaging Reporting and Data System; CI = confidence interval; PPV = positive predictive value; NPV = negative predictive value. Positive likelihood ratio = sensitivity/(1 − specificity). Negative likelihood ratio = (1 − sensitivity)/specificity. Diagnostic odds ratio = (true positive × true negative)/(false positive × false negative). Youden index = sensitivity + specificity − 1. PPV, NPV, and accuracy were calculated from the observed surgically enriched study cohort and should not be extrapolated directly to unselected thyroid nodule populations.

Diagnostic measure	ACR TI-RADS TR4 or higher	EU-TI-RADS ≥4
Test-positive definition	TR4 or TR5	Category 4 or 5
True positive	131	130
False positive	7	4
True negative	127	130
False negative	19	20
Sensitivity	131/150 (87.3%; 95% CI: 81.1%-91.7%)	130/150 (86.7%; 95% CI: 80.3%-91.2%)
Specificity	127/134 (94.8%; 95% CI: 89.6%-97.4%)	130/134 (97.0%; 95% CI: 92.6%-98.8%)
Positive predictive value	131/138 (94.9%; 95% CI: 89.9%-97.5%)	130/134 (97.0%; 95% CI: 92.6%-98.8%)
Negative predictive value	127/146 (87.0%; 95% CI: 80.6%-91.5%)	130/150 (86.7%; 95% CI: 80.3%-91.2%)
Accuracy	258/284 (90.8%; 95% CI: 86.9%-7%)	260/284 (91.5%; 95% CI: 87.7%-94.3%)
Positive likelihood ratio	16.72 (95% CI: 8.11-34.48)	29.03 (95% CI: 11.04-76.38)
Negative likelihood ratio	0.13 (95% CI: 0.09-0.20)	0.14 (95% CI: 0.09-0.21)
Diagnostic odds ratio	125.1 (95% CI: 50.8-307.8)	211.3 (95% CI: 70.3-635.1)
Youden index	0.821	0.837

Overall, EU-TI-RADS demonstrated slightly higher specificity, whereas ACR TI-RADS showed marginally higher sensitivity, indicating a modest trade-off between false-positive and false-negative classifications. These small differences reflect distinct false-positive and false-negative classification profiles rather than a statistically significant difference in overall discriminatory performance and should be interpreted in the context of the surgically enriched study cohort.

Confusion matrix analysis

Confusion matrix analysis for the clinically relevant thresholds (ACR TI-RADS TR4 or higher and EU-TI-RADS ≥ 4) is displayed within Table 3, presenting true-positive, false-positive, true-negative, and false-negative counts alongside the corresponding diagnostic performance metrics.

Using the case-sensitive HC number field as the patient identifier, the operated-cohort dataset included 284 nodules from 233 patients. Overall, 182 patients contributed one operated nodule, and 51 patients contributed two operated nodules; no patient contributed more than two nodules (Appendix, Supplementary Table S8).

A single-index-nodule sensitivity analysis was performed after restricting the dataset to one nodule per patient. This analysis included 233 index nodules, of which 129 were malignant and 104 were benign. ACR TI-RADS TR4 or higher yielded 112 true-positive, 5 false-positive, 99 true-negative, and 17 false-negative classifications, corresponding to a sensitivity of 86.8%, a specificity of 95.2%, PPV of 95.7%, NPV of 85.3%, and an accuracy of 90.6%. EU-TI-RADS ≥4 yielded 111 true-positive, 2 false-positive, 102 true-negative, and 18 false-negative classifications, corresponding to a sensitivity of 86.0%, a specificity of 98.1%, PPV of 98.2%, NPV of 85.0%, and an accuracy of 91.4% (Appendix, Supplementary Table S9). These results were consistent with the primary nodule-level analysis.

Diagnostic contribution of individual ultrasound features

The diagnostic contribution of individual ACR TI-RADS component criteria is summarized in Table 4. Among the evaluated features, echogenicity (AUC: 0.857; 95% CI: 0.810-0.905; p < 0.001) and echogenic foci/microcalcifications (AUC: 0.820; 95% CI: 0.768-0.873; p < 0.001) showed the strongest individual diagnostic contribution, followed by composition (AUC: 0.756; 95% CI: 0.694-0.818; p < 0.001) and margin (AUC: 0.721; 95% CI: 0.660-0.783; p < 0.001). In contrast, orientation, corresponding to the ACR shape criterion of wider-than-tall versus taller-than-wide, demonstrated limited standalone discrimination (AUC: 0.556; 95% CI: 0.486-0.627; p = 0.12). The complete feature-level analysis is provided in the Appendix (Supplementary Table S7), with corresponding curves shown in Figure 3.

**Table 4:** Receiver operating characteristic analysis of the selected ACR TI-RADS component criteria and additional ultrasound features for predicting malignant versus benign histopathology in surgically excised thyroid nodules (n = 284) ROC analysis was performed using the original ordinal categorical coding for each ACR TI-RADS component criterion and for the two features inversely associated with malignancy (nodule size and comet-tail artifact). Criterion 3 corresponds to the shape/orientation component (wider-than-tall versus taller-than-wide) as defined in the ACR TI-RADS classification. AUC values shown are consistent with those reported in the Appendix (Supplementary Table S7). ACR TI-RADS = American College of Radiology Thyroid Imaging Reporting and Data System; CI = confidence interval; NS = not significant

Ultrasound criterion or feature	AUC	95% CI	P value
Criterion 2: Echogenicity	0.857	0.810-0.905	<0.001
Criterion 5: Echogenic foci (microcalcification)	0.82	0.768-0.873	<0.001
Criterion 1: Composition	0.756	0.694-0.818	<0.001
Criterion 4: Margin	0.721	0.660-0.783	<0.001
Criterion 3: Shape (orientation)	0.556	0.486-0.627	0.12 (NS)
Nodule size (inverse association)	0.695	0.631-0.760	<0.001
Comet-tail artifact (inverse association)	0.58	0.509-0.652	0.028

**Figure 3:**
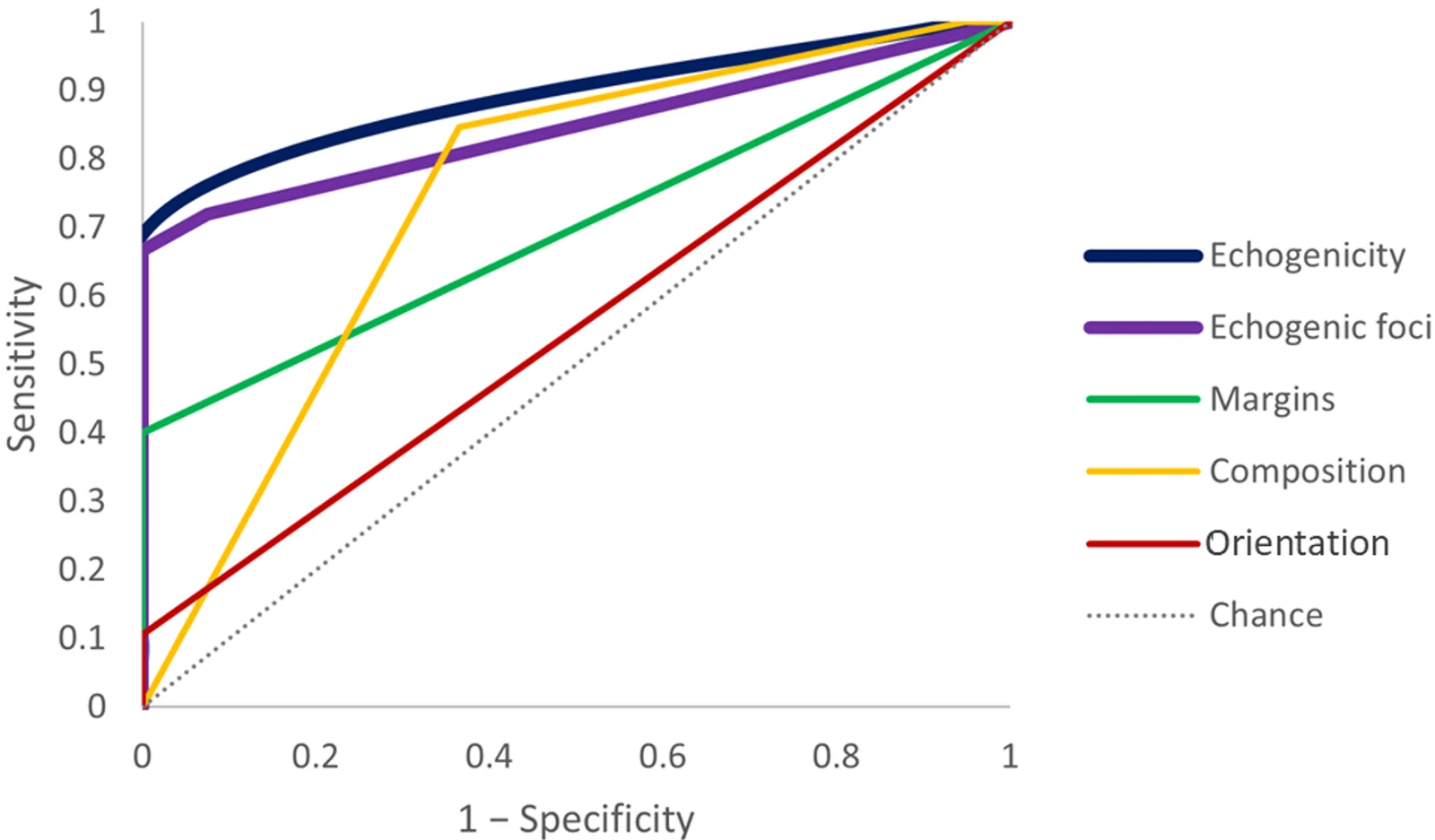
Receiver operating characteristic curves illustrating the diagnostic performance of the selected ultrasound criteria, including echogenicity, echogenic foci, margins, composition, and orientation, for discriminating malignant from benign thyroid nodules in a histopathology-validated surgical cohort of 284 nodules The X-axis represents 1 - specificity, the Y-axis represents sensitivity, and the dashed diagonal line represents chance performance.

Subgroup and illustrative findings

Subgroup analyses stratified by histological malignant subtype are provided in the Appendix. In the papillary thyroid carcinoma subgroup (n = 255; 121 papillary carcinomas and 134 benign nodules), all three classification approaches demonstrated excellent discriminatory performance, with AUCs ranging from 0.968 to 0.973 (Appendix, Supplementary Table S5). In the non-papillary malignancy subgroup (n = 163; 29 non-papillary malignancies and 134 benign nodules), discrimination was lower and more variable, with AUCs ranging from 0.719 to 0.819 (Appendix, Supplementary Table S6). These non-papillary subgroup estimates should be interpreted cautiously because of the small number of non-papillary malignant nodules. This lower and more variable discrimination for non-papillary malignancies is consistent with the histopathology-based analysis by Hekimsoy et al., in which follicular-patterned lesions - including follicular-variant papillary carcinoma and non-invasive follicular thyroid neoplasm with papillary-like nuclear features (NIFTP) - characteristically lacked classic high-suspicion sonographic features and were under-stratified by both systems, such that reclassifying NIFTP as benign improved the measured diagnostic performance of ACR TI-RADS and EU-TI-RADS [17]. Consistent with this, follicular-patterned carcinomas accounted for most false-negative classifications in our cohort, including 13 of 19 for ACR TI-RADS and 14 of 20 for EU-TI-RADS (Appendix, Supplementary Table S10), underscoring a limitation of ultrasound risk stratification for some follicular-patterned malignancies. Clinically, these findings emphasize that a low-risk sonographic appearance should not independently exclude malignancy in follicular-patterned lesions and that TI-RADS classification should be interpreted alongside cytological findings and the overall clinical context when determining further management.

Representative ultrasound cases illustrating high-risk features and diagnostic limitations are shown in Figure 4 and the Appendix (Supplementary Figures S2 and S3).

**Figure 4:**
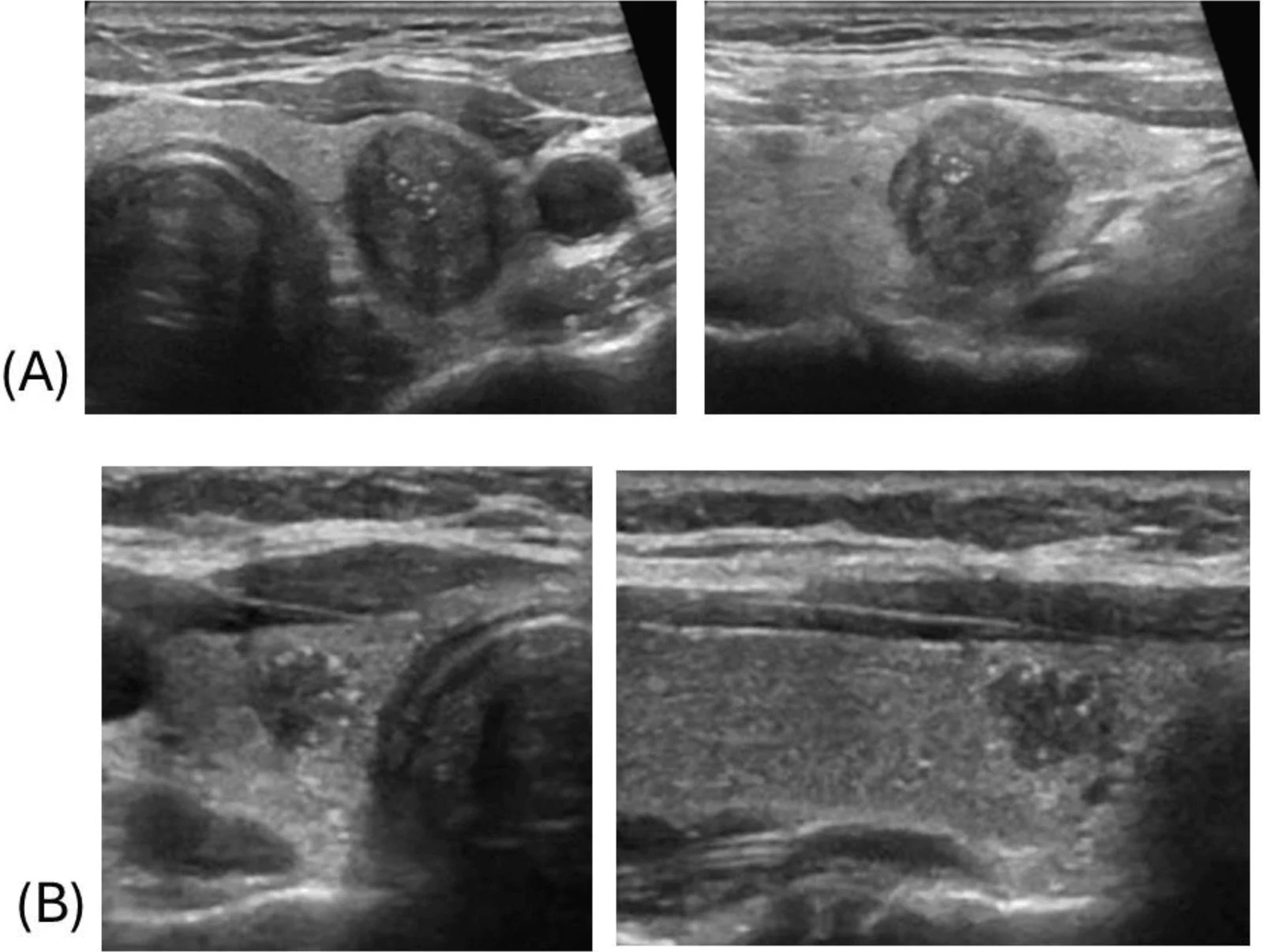
Representative anonymized ultrasound images of high-risk thyroid nodules classified as ACR TI-RADS 5 and EU-TI-RADS 5, with surgical histopathological confirmation of malignancy (A) A 42-year-old female presenting with a palpable left thyroid nodule. Transverse and sagittal images demonstrate a solid hypoechoic nodule in the left thyroid lobe with punctate echogenic foci and taller-than-wide orientation (histopathology: papillary thyroid carcinoma). (B) A 36-year-old female referred with an incidental sonographic finding. Transverse and sagittal images of a small 0.8 cm right thyroid lobe nodule demonstrate a solid hypoechoic nodule with microcalcifications and irregular margins (histopathology: papillary thyroid carcinoma). Images are original anonymized ultrasound images from the study dataset. No generative artificial-intelligence tools were used to create or modify the images. ACR TI-RADS = American College of Radiology Thyroid Imaging Reporting and Data System; EU-TI-RADS = European Thyroid Imaging Reporting and Data System

## Discussion

Principal findings

In this histopathology-validated analysis of 284 surgically excised thyroid nodules from 233 patients, ACR TI-RADS and EU-TI-RADS achieved excellent and broadly comparable diagnostic performance (AUC: 0.948 versus 0.933; ΔAUC: 0.015 with bootstrap 95% CI crossing zero), distinguished less by discriminatory power than by classification-error profile. At the clinically relevant thresholds of ACR TI-RADS TR4 or higher and EU-TI-RADS ≥ 4, ACR TI-RADS produced slightly fewer false-negative classifications (19 versus 20), whereas EU-TI-RADS produced fewer false-positive classifications (4 versus 7). Feature-level ROC analysis showed that echogenicity and echogenic foci/microcalcifications provided the strongest individual discrimination, while composition and margin showed intermediate univariate discriminatory contribution. Orientation, separately coded nodule shape, nodule size, and comet-tail artifact showed weaker or cohort-dependent discrimination.

What this study adds

This work integrates several methodological elements within a single histopathology-validated Gulf-region cohort: threshold-based accuracy metrics with Wilson 95% confidence intervals, full confusion-matrix profiling, feature-level ROC analysis, subgroup analysis by malignant histological subtype, and assessment of potential clustering effects through cluster-size characterization and a single-index-nodule sensitivity analysis. The integrated approach was designed to close specific reporting gaps identified in prior comparative TI-RADS studies and to provide clinically actionable feature-level and error-profile information for surgically enriched populations.

The present analysis is a distinct secondary study within the same PHCC program as our previously published investigation of 809 nodules, which evaluated guideline-based fine-needle aspiration recommendations using a composite reference standard [15]. While the earlier study assessed biopsy-decision performance in a broader definitive-outcome cohort with a malignancy prevalence of 19.4%, the current analysis is restricted to the 284 surgically excised nodules within that programme, uses postoperative histopathology exclusively as the reference standard, and addresses complementary questions concerning intrinsic discrimination, feature contribution, classification-error profiles, histological subgroup performance, and the potential influence of within-patient clustering. The present study does not reproduce tables, figures, ROC curves, or diagnostic performance estimates from the previous publication. It provides a distinct secondary analysis of the surgically excised, histopathology-validated subset. Together, they illustrate that intrinsic discriminatory power and biopsy-decision efficiency are related but distinct constructs, and they can behave differently across selection strata even when the same TI-RADS lexicon is applied.

Comparison with prior histopathology-verified cohorts

The high AUC estimates observed in the present cohort are broadly in line with prior histopathology-verified comparisons reporting strong performance of both systems. Magri et al. reported comparable diagnostic performance between ACR TI-RADS and EU-TI-RADS in 304 nodules from 255 thyroidectomised patients, with similar AUC values and no statistically significant difference in discriminatory performance [18]. A histopathology-based study of 251 nodules in 165 patients also reported comparable sensitivity and specificity between the two systems, supporting broadly similar diagnostic performance [17]. Chatti et al. and subsequent meta-analytic reports have similarly concluded that ACR TI-RADS and EU-TI-RADS deliver broadly equivalent risk stratification when applied within histopathology-verified cohorts, with cohort-specific differences in specificity attributable to spectrum enrichment [7,9-12]. The modestly higher EU-TI-RADS specificity observed in this cohort should be interpreted in the context of enrichment for surgically resected lesions rather than as evidence of intrinsic system superiority.

Feature-level interpretation

Echogenicity and microcalcifications were the two dominant features across every analysis, aligning with the pathobiology of papillary thyroid carcinoma and with contemporary meta-analytic evidence [2-4]. Composition and margin contributed intermediate signal, and none of the individual features achieved the discriminatory performance observed for the integrated TI-RADS classifications, supporting the rationale underlying composite risk stratification systems [5,6]. In this surgically excised cohort, microcalcifications were present in 95 malignant and no benign nodules, taller-than-wide orientation in 16 malignant and no benign nodules, very hypoechoic echotexture in 12 malignant and no benign nodules, and irregular or lobulated margins in 58 malignant and no benign nodules (Appendix, Supplementary Table S11). Orientation, nodule shape, size, and comet-tail findings. Orientation did not reach statistical significance in this cohort (AUC: 0.556; p = 0.12). The separately coded nodule-shape variable also showed limited discrimination (AUC: 0.541; p = 0.26; Appendix, Supplementary Table S7). The limited standalone discrimination of orientation should be interpreted cautiously because taller-than-wide orientation was uncommon in this cohort, occurring in only 16 of 284 nodules. This finding should not be generalized beyond the surgical spectrum evaluated here. Nodule size showed an inverse association with malignancy (AUC: 0.695). This association may reflect surgical-selection and spectrum effects, whereby the indications leading to surgery may differ between smaller sonographically suspicious nodules and larger low-risk nodules. Because surgical indication was not evaluated as an explanatory variable, the observed association should not be interpreted as evidence that smaller nodules are intrinsically more likely to be malignant. Comet-tail artifact also showed an inverse association with malignancy (AUC: 0.580), consistent with its recognized association with benign colloid nodules.

Clinical implications

Both ACR TI-RADS and EU-TI-RADS demonstrated excellent diagnostic performance in this surgically enriched cohort. EU-TI-RADS produced fewer false-positive classifications, whereas ACR TI-RADS produced one fewer false-negative classification at the evaluated thresholds. These small differences may inform local operational considerations, but they should not be interpreted as establishing the clinical superiority of either system.

Feature-level analysis converges on echogenicity and microcalcifications as the most influential individual descriptors, supporting targeted training investment in their reproducible identification. The observed inverse association between nodule size and malignancy should not be extrapolated to routine practice and highlights the importance of pairing TI-RADS classification with clinical judgment when interpreting large but sonographically low-risk nodules.

Strengths

The principal strengths are the use of postoperative histopathology as the reference standard, blinded reassessment by three consultant radiologists with consensus resolution of disagreements, integrated reporting of ROC, threshold-based, confusion-matrix, feature-level, and subgroup metrics with STARD-aligned participant flow, and evaluation of the potential influence of within-patient clustering through cluster-size characterization and a single-index-nodule sensitivity analysis. Several limitations should be acknowledged and are outlined in the following section.

Limitations

Several limitations should be considered. First and most importantly, this was a retrospective single-country study conducted within a surgically selected cohort, with a malignancy prevalence (52.8%) substantially higher than that observed in unselected thyroid nodule populations (5-15%). Because this surgically enriched cohort had a malignancy prevalence of 52.8%, predictive values should not be extrapolated to unselected screening or surveillance populations. Sensitivity, specificity, and AUC may also differ in unselected populations because of spectrum and verification bias. In addition, the specific indication for surgery was not systematically coded for each nodule; consequently, the relative frequencies of suspicious or indeterminate preoperative findings, compressive or cosmetic symptoms, multinodular disease, patient preference, and multidisciplinary recommendations could not be quantified, particularly among nodules ultimately shown to be benign. Second, formal interobserver agreement analysis was not performed. Although ultrasound images were re-evaluated by three consultant radiologists using standardized criteria and blinded to histopathology, final analytic categories were assigned by consensus, and the reproducibility of individual ultrasound features and TI-RADS classifications could not be quantified. Future prospective studies incorporating independent blinded reassessment by multiple readers, with formal kappa and intraclass correlation coefficient analysis, are warranted. A prior histopathology-based study reported high interobserver reproducibility for both systems [17]. However, those results cannot substitute for reader-specific agreement assessment in the present cohort, and the reproducibility of the current classifications remains unquantified. Third, adjusted multivariable feature-level analysis was not feasible for the reasons described in the "Statistical Analysis" subsection; this limitation may be addressed in future studies using larger and less-enriched cohorts and appropriate penalized regression methods. Fourth, ultrasound examinations were performed on multiple scanners over the study period; although this reflects real-world clinical practice, machine-specific effects cannot be fully excluded. Fifth, the relatively small number of non-papillary malignant nodules limits statistical precision for histotype-specific subgroup analyses. Sixth, external validation in multicenter prospective cohorts across different scanners, operators, and healthcare systems is required to confirm reproducibility.

## Conclusions

ACR TI-RADS and EU-TI-RADS demonstrated excellent and broadly comparable histopathology-validated diagnostic performance in this surgically enriched cohort. Their differences primarily reflected distinct classification-error profiles rather than clear superiority of either system. Echogenicity and echogenic foci provided the strongest individual discriminatory contributions, while follicular-patterned malignancies accounted for most false-negative classifications. Prospective multicenter validation in unselected thyroid nodule populations is warranted.

## Figures and Tables

**Figure 5:**
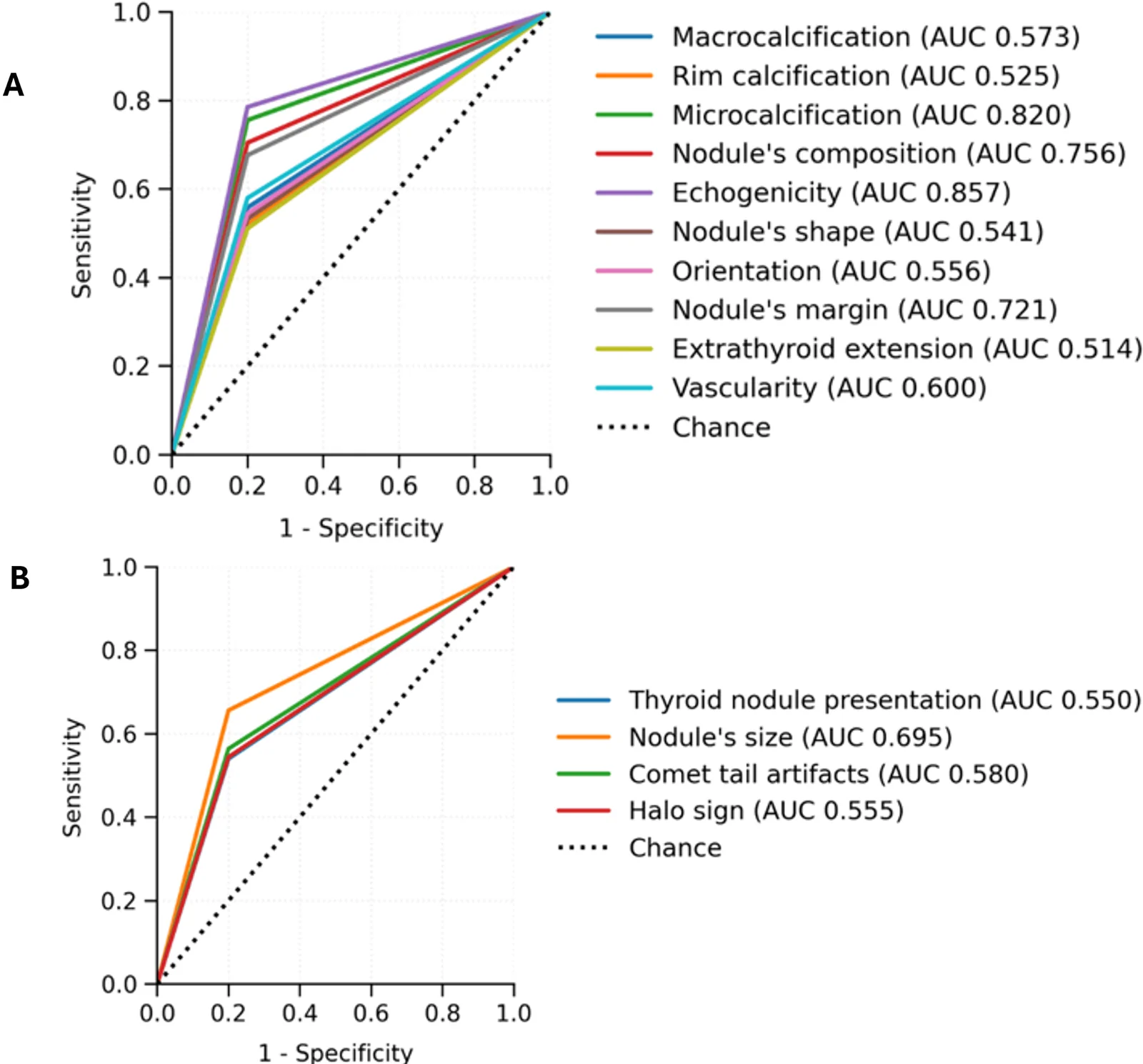
Receiver operating characteristic (ROC) curves illustrating the diagnostic performance of selected individual ultrasound features for predicting malignant versus benign histopathology in surgically excised thyroid nodules (A) ROC curves for grayscale and Doppler ultrasound features, including echogenicity, echogenic foci (microcalcifications), composition, margin characteristics, orientation, macrocalcifications, rim calcifications, extrathyroidal extension, and vascularity. (B) ROC curves for additional ultrasound features, including thyroid nodule presentation (solitary vs multiple nodules), nodule size, comet-tail artifacts, and halo sign. The dashed diagonal line represents chance performance.

**Figure 6:**
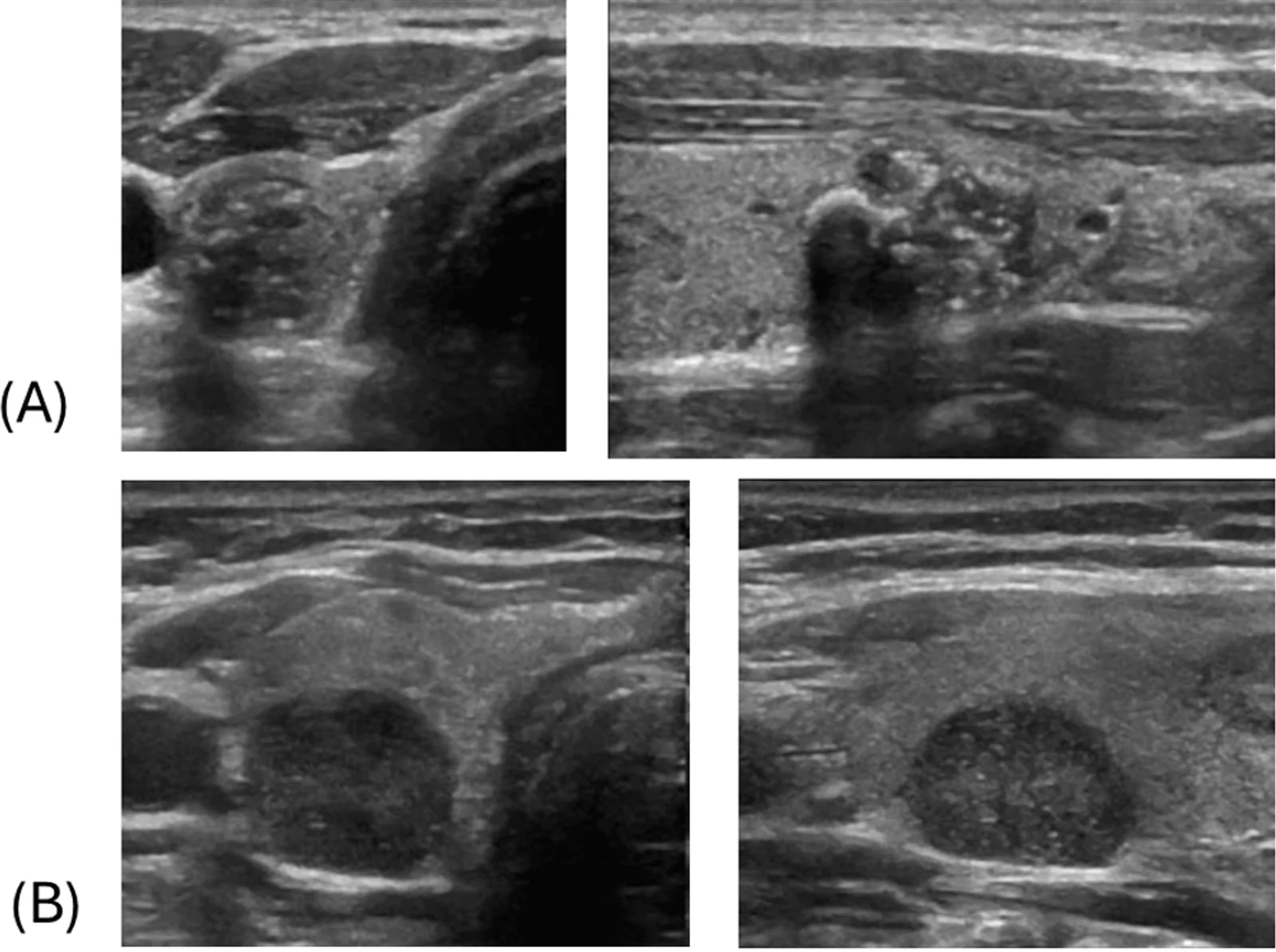
Representative anonymized ultrasound images of malignant thyroid nodules confirmed by surgical histopathology, demonstrating varied high-suspicion sonographic features (A) Transverse and sagittal grayscale images demonstrate a solid hypoechoic nodule with lobulated margins, microcalcifications, and macrocalcification. (B) Transverse and sagittal grayscale images demonstrate a solid, markedly hypoechoic nodule with punctate echogenic foci. Surgical histopathology confirmed papillary thyroid carcinoma in both cases.

**Figure 7:**
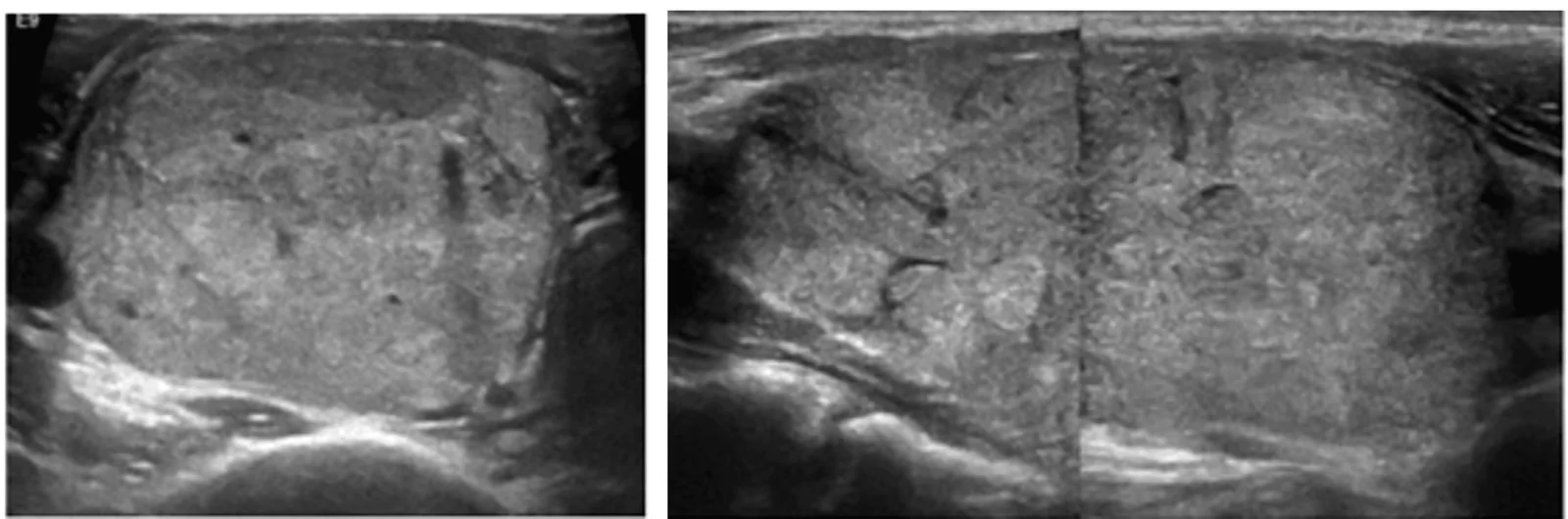
Transverse and sagittal grayscale images demonstrate a 5.2 cm, nearly completely solid isoechoic nodule with smooth margins and no high-suspicion features, classified as ACR TI-RADS 3 and EU-TI-RADS 3 Surgical histopathology confirmed encapsulated angioinvasive follicular thyroid carcinoma. These images illustrate false-negative risk category classification of follicular-patterned malignancy. ACR TI-RADS = American College of Radiology Thyroid Imaging Reporting and Data System; EU-TI-RADS = European Thyroid Imaging Reporting and Data System

## Appendices

Appendix 1

Supplementary Figure S1

Supplementary Figure S2

Supplementary Figure S3

Supplementary Table S1

**Table 5:** Full breakdown of ultrasound characteristics in surgically excised thyroid nodules (n = 284) Nodule shape (oval, round, or irregular) and nodule orientation (wider-than-tall or taller-than-wide) were recorded as separate sonographic variables. Their identical marginal distributions in this cohort reflect the observed data and do not indicate duplicate coding. Percentages are calculated using the total number of surgically excised nodules; patient-level characteristics (age, sex) are reported at the patient level (n = 233) in Table 1 of the main manuscript.

Domain	Category	n	%
Nodule site	Right lobe	130	45.8
	Left lobe	136	47.9
	Isthmus	18	6.3
Maximum size category	<0.5 cm	0	0
	0.5-<1.0 cm	14	4.9
	1.0-<1.5 cm	47	16.5
	1.5-2.0 cm	28	9.9
	>2.0-<2.5 cm	20	7
	≥2.5 cm	175	61.6
Composition	Spongiform	0	0
	Cystic	2	0.7
	Almost entirely cystic	5	1.8
	Mixed cystic - solid	101	35.6
	Almost entirely solid	56	19.7
	Solid	120	42.3
Echogenicity	Anechoic	7	2.5
	Isoechoic	161	56.7
	Hyperechoic	3	1.1
	Hypoechoic	101	35.6
	Very (markedly) hypoechoic	12	4.2
Shape	Oval	268	94.4
	Round	5	1.8
	Irregular	11	3.9
Orientation	Wider-than-tall	268	94.4
	Taller-than-wide	16	5.6
Margins	Smooth	150	52.8
	Ill-defined	76	26.8
	Lobulated	30	10.6
	Irregular	28	9.9
Echogenic foci/calcifications	Microcalcifications present	95	33.5
	Macrocalcifications present	42	14.8
	Rim calcifications present	7	2.5
Extrathyroidal extension	Absent	280	98.6
	Capsular bulge	4	1.4
	Overt extrathyroidal extension	0	0
Vascularity (Doppler)	Not assessed/NA	20	7
	Type I (no peri-/intranodular flow)	24	8.5
	Type II (peri- and/or slight intranodular flow)	174	61.3
	Type III (marked intranodular flow ± slight perinodular flow)	66	23.2
Halo sign	Absent	267	94
	Present	17	6
Comet–tail artifact	Absent	253	89.1
	Present	31	10.9

Supplementary Table S2

**Table 6:** Cross-tabulation of ACR TI-RADS categories versus final histopathological outcomes in surgically excised thyroid nodules (n = 284) Row totals and category-specific malignancy rates are added for transparency. ACR TI-RADS = American College of Radiology Thyroid Imaging Reporting and Data System

ACR TI-RADS category	Benign (n)	Malignant (n)	Total (n)	Malignancy rate (%)
TR1	7	0	7	0
TR2	72	6	78	7.7
TR3	48	13	61	21.3
TR4	7	29	36	80.6
TR5	0	102	102	100
Total	134	150	284	52.8

Supplementary Table S3

**Table 7:** Cross-tabulation of EU-TI-RADS categories versus the final histopathological outcomes in surgically excised thyroid nodules (n = 284) Row totals and category-specific malignancy rates are added for transparency. EU-TI-RADS = European Thyroid Imaging Reporting and Data System

EU-TI-RADS category	Benign (n)	Malignant (n)	Total (n)	Malignancy rate (%)
EU-2 (benign)	7	0	7	0
EU-3 (low risk)	123	20	143	14
EU-4 (intermediate risk)	4	15	19	78.9
EU-5 (high risk)	0	115	115	100
Total	134	150	284	52.8

Supplementary Table S4

**Table 8:** Final histopathological diagnoses of surgically excised thyroid nodules (n = 284)

Diagnosis	Number (%)
Benign	134 (47.2%)
Malignant	150 (52.8%)
Papillary	121 (42.6%)
Other malignancies	29 (10.2%)

Supplementary Table S5

**Table 9:** Diagnostic performance (ROC analysis) of ACR TI-RADS and EU-TI-RADS for predicting papillary thyroid carcinoma versus benign histopathology ROC analysis was performed using histopathology as the reference standard. Papillary thyroid carcinomas were coded as the positive outcome. Analysis restricted to 255 nodules (121 papillary thyroid carcinomas + 134 benign nodules). Non-papillary malignancies were excluded. CI = confidence interval; ACR TI-RADS = American College of Radiology Thyroid Imaging Reporting and Data System; EU-TI-RADS = European Thyroid Imaging Reporting and Data System

Classification System	AUC	95% CI	P value
ACR TI-RADS point score	0.973	0.950-0.996	<0.001
EU-TI-RADS categories	0.972	0.949-0.996	<0.001
ACR TI-RADS categories	0.968	0.943-0.992	<0.001

Supplementary Table S6

**Table 10:** Diagnostic performance (ROC analysis) of ACR TI-RADS and EU-TI-RADS for predicting non-papillary thyroid malignancies versus benign histopathology ROC analysis was performed using histopathology as the reference standard. Non-papillary thyroid malignancies were coded as the positive outcome. Results should be interpreted cautiously because of the limited number of non-papillary malignant nodules. Analysis restricted to 163 nodules (29 non-papillary malignancies + 134 benign nodules). Papillary carcinomas were excluded. CI = confidence interval; ACR TI-RADS = American College of Radiology Thyroid Imaging Reporting and Data System; EU-TI-RADS = European Thyroid Imaging Reporting and Data System

Classification System	AUC	95% CI	P value
ACR TI-RADS point score	0.819	0.723-0.915	<0.001
ACR TI-RADS categories	0.761	0.611-0.911	<0.001
EU-TI-RADS categories	0.719	0.594-0.844	<0.001

Supplementary Table S7

**Table 11:** Receiver operating characteristic (ROC) analysis of individual ultrasound features for predicting malignant versus benign histopathology in surgically excised thyroid nodules (n = 284) ROC analysis was performed for each ultrasound feature using its original categorical coding. For binary variables, direction of test positivity was specified as present versus absent. CI = confidence interval; NS = not significant

Ultrasound Feature	AUC	95% CI	P value
Features positively associated with malignancy			
Echogenicity	0.857	0.810-0.905	<0.001
Microcalcification (echogenic foci)	0.82	0.768-0.873	<0.001
Composition	0.756	0.694-0.818	<0.001
Margin	0.721	0.660-0.783	<0.001
Vascularity	0.6	0.530-0.669	0.006
Macrocalcification	0.573	0.503-0.643	0.045
Orientation (ACR shape criterion)	0.556	0.486-0.627	0.12 (NS)
Nodule shape (oval/round/irregular)	0.541	0.470-0.612	0.26 (NS)
Rim calcification	0.525	0.453-0.596	0.50 (NS)
Extrathyroidal extension	0.514	0.443-0.585	0.70 (NS)
Features inversely associated with malignancy			
Nodule size (cm)	0.695	0.631-0.760	<0.001
Comet-tail artifact	0.58	0.509-0.652	0.028
Halo sign	0.555	0.483-0.627	0.13 (NS)
Thyroid nodule presentation (solitary vs multiple)	0.55	0.479-0.621	0.17 (NS)

Supplementary Table S8

**Table 12:** Cluster-size distribution in the operated cohort Cluster size was calculated. No patient contributed more than two surgically excised nodules.

Number of operated nodules per patient	Patients, n (%)	Nodules contributed, n (%)
1	182/233 (78.1%)	182/284 (64.1%)
2	51/233 (21.9%)	102/284 (35.9%)
Total	233/233 (100%)	284/284 (100%)

Supplementary Table S9

**Table 13:** Single-index-nodule sensitivity analysis in the operated cohort The single-index-nodule analysis was restricted to one nodule per patient. The index nodule was selected according to the hierarchy described in the Methods section: highest TI-RADS category first and largest diameter if categories were tied. TP = true positive; FP = false positive; TN = true negative; FN = false negative; PPV = positive predictive value; NPV = negative predictive value; ACR TI-RADS = American College of Radiology Thyroid Imaging Reporting and Data System; EU-TI-RADS = European Thyroid Imaging Reporting and Data System

System/threshold	TP	FP	TN	FN	Sensitivity, % (95% CI)	Specificity, % (95% CI)	PPV, % (95% CI)	NPV, % (95% CI)	Accuracy, % (95% CI)
ACR TI-RADS TR4 or higher	112	5	99	17	86.8 (79.9-91.6)	95.2 (89.2-97.9)	95.7 (90.4-98.2)	85.3 (77.8-90.6)	90.6 (86.1-93.7)
EU-TI-RADS ≥4	111	2	102	18	86.0 (79.091.0)	98.1 (93.3-99.5)	98.2 (93.8-99.5)	85.0 (77.5-90.3)	91.4 (87.1-94.4)

Supplementary Table S10

**Table 14:** Histological distribution of missed (false-negative) malignant nodules for ACR TI-RADS and EU-TI-RADS at the clinically relevant classification thresholds (ACR < TR4 and EU < 4) in the operated cohort (n = 284) False-negative nodules were histopathologically confirmed malignancies classified below the prespecified risk-category threshold used in this analysis: ACR TI-RADS < TR4 or EU-TI-RADS <4. These thresholds were used for binary diagnostic classification and should not be interpreted as size-independent fine-needle aspiration thresholds. Counts were derived directly from the operated-cohort dataset and reproduce the false-negative totals reported in Table 3 (19 for ACR TI-RADS and 20 for EU-TI-RADS). Follicular-pattern carcinomas accounted for most missed malignancies in both systems (13/19 (68.4%) for ACR TI-RADS and 14/20 (70.0%) for EU-TI-RADS), whereas papillary thyroid carcinoma accounted for a minority (six in each system). The single additional EU-TI-RADS false-negative corresponds to one minimally invasive follicular carcinoma classified as ACR TR4 (correctly captured) but EU-3 (missed). The predominance of follicular-patterned carcinomas among false-negative classifications is consistent with their less frequent expression of classic high-suspicion ultrasound features and is illustrated in Supplementary Figure S3. ACR TI-RADS = American College of Radiology Thyroid Imaging Reporting and Data System; EU-TI-RADS = European Thyroid Imaging Reporting and Data System

Histological pattern of missed (false-negative) malignancy	ACR TI-RADS < TR4 (n = 19)	EU-TI-RADS < 4 (n = 20)
Follicular thyroid carcinoma (classic)	5	5
Minimally invasive follicular carcinoma	6	7
Encapsulated angioinvasive follicular carcinoma	1	1
Encapsulated follicular carcinoma	1	1
Follicular-pattern subtotal	13	14
Papillary thyroid carcinoma	6	6
Total false-negative malignant nodules	19	20

Supplementary Table S11

**Table 15:** Cross-tabulation of the selected high-suspicion ultrasound features against the final histopathology in surgically excised thyroid nodules (n = 284) Final postoperative histopathology was used as the reference standard. The operated cohort comprised 134 benign and 150 malignant nodules. Microcalcifications, taller-than-wide orientation, very hypoechoic echotexture, and irregular or lobulated margins were not observed in any benign nodule in this surgically selected cohort. The irregular-or-lobulated margin category combines 30 nodules with lobulated margins and 28 nodules with irregular margins. The statistical implications of these zero cells are described in the "Statistical Analysis" subsection.

High-suspicion ultrasound feature	Benign with feature present, n	Malignant with feature present, n	Benign with feature absent, n	Malignant with feature absent, n
Microcalcifications present	0	95	134	55
Taller-than-wide orientation	0	16	134	134
Very hypoechoic echotexture	0	12	134	138
Irregular or lobulated margins	0	58	134	92
